# Tubal Endometriosis: From Bench to Bedside, A Scoping Review

**DOI:** 10.3390/jpm12030362

**Published:** 2022-02-26

**Authors:** Anastasia Prodromidou, Nikolaos Kathopoulis, Dimitrios Zacharakis, Themos Grigoriadis, Ioannis Chatzipapas, Athanasios Protopapas

**Affiliations:** 1st Department of Obstetrics & Gynecology, Medical School, National and Kapodistrian University of Athens, “Alexandra” Hospital, Lourou 2, 11528 Athens, Greece; nickatho@gmail.com (N.K.); dimzac@hotmail.com (D.Z.); tgregos@yahoo.com (T.G.); ixatzipapas@yahoo.gr (I.C.); prototha@otenet.gr (A.P.)

**Keywords:** tubal endometriosis, fallopian tube, endometriosis, prevalence

## Abstract

Tubal endometriosis (EM) refers to the detection of ectopic endometrial implants on tubes. It may cause a significant defect of the tubes, translating into dysmenorrhea, pelvic pain, and infertility. We aimed to evaluate the disease characteristics, prevalence, histopathological findings and genetic profile of patients with tubal EM. A thorough search of three electronic databases was performed for studies that presented outcomes of patients with tubal EM. Thirteen studies (four observational, seven case reports, two genetic) were considered eligible for inclusion. The prevalence of tubal EM ranged from 6.9% to 69%. The predominant symptoms for referral of patients were infertility and abdominal pain. Women of reproductive age underwent salpingectomy for the management of the disease. Only one case of malignant transformation was recorded in a 60-year-old patient. The prevalence of tubal EM ranges depending on the indication for surgery, the presence of concomitant pelvic EM and the type of diagnosis and treatment. Further, more extensive, larger studies are warranted to evaluate the impact of tubal EM in the progression and prognosis of EM, the effect of salpingectomy in the improvement of disease-related symptoms and to designate the group of patients that could benefit from risk-reducing salpingectomy based on the risk of developing ovarian malignancy.

## 1. Introduction

Endometriosis (EM) is a chronic benign gynecological disease, which is defined as the presence of endometrial deposits outside the uterine cavity [1]. The estimated prevalence of the disease is approximately 10% among women of reproductive age [2]. Endometriosis is most commonly identified in the pelvis and it affects the ovaries, the pelvic peritoneum cul-de-sac and uterosacral ligaments [3]. Additionally, less common extrapelvic endometriosis sites in the gastrointestinal and urinary tract, chest and brain have also been recorded, while there are also reports of multiple endometriosis sites especially in patients with deep infiltrating (DIE) endometriosis in as high as 44% of them [3,4]. Pain and infertility are the most common primary symptoms encountered in 30–50% of women with EM [5]. Retrograde menstruation, firstly described by Sampson et al., has been considered as the most prevalent theory for the pathogenesis of EM [2]. Women with obstructive outflow diseases are considered more susceptible to retrograde menstrual flow, which could facilitate the transportation of endometriotic menstrual cells to the peritoneal cavity through the fallopian tubes [2]. The genetic and epigenetic theory, according to which already existing endometrial cells are modified and result in the development of the clinical manifestation of the disease, could explain why not all women with retrograde menstruation will develop EM [6]. Coelomic metaplasia is another theory that supports the transformation of peritoneal, pleural and ovarian mesothelial cells to endometriosis, while theories about the lymphatic and vascular spread of endometrial cells are still under investigation [7]. Treatment options may range from conservative medication hormonal-based treatment to more invasive surgical procedures. Despite the benign nature of the disease, the risk of malignant transformation reaches a proportion of approximately 1% [8]. History of EM is related to a significantly elevated risk of developing ovarian cancer [8]. The most common histological subtypes arising from EM are endometrioid adenocarcinoma, clear cell carcinoma and low-grade serous carcinoma [8,9]. Histopathologically, the malignant transformation is recognized as cytologic atypia and architectural proliferation [10]. Cytologic atypia is defined as the transition from benign EM to carcinoma and is classified as moderate (simple hyperplasia or cellular atypia) or severe (complex hyperplasia or cellular atypia that is more evident) [11]. Concerning cellular proliferation, complex hyperplasia is translated into glandular proliferation and reduced stroma, which can evolve towards ovarian cancer [11]. As mentioned above, endometrioid carcinoma or clear cell carcinoma are the most prevalent types, and their gross appearance consists of the typical histology of each type of malignancy including cribiform, glandular or solid architecture and papillary, solid or tubulocystic architecture for each cancer type, respectively [11]. Mitoses are also detected in both types. Endometriosis associated ovarian cancer (EAOC) is defined as the coexistence of malignant cells and EM either in the same ovary or EM in the one and cancer in the other ovary [10,11]. 

Tubal EM is defined as the detection of ectopic endometrial implants on the tubes. It may cause a significant defect of the fallopian tubes and functional and structural disorders, which may translate into dysmenorrhea, pelvic pain and infertility. Notwithstanding the multiple reports on the potential contribution of tubal EM on the pathogenesis of endometriosis related symptomatology, the exact aspects of the disease still remain elusive. 

We performed a scoping review aiming to accumulate the currently available literature on tubal EM with special consideration to disease characteristics, prevalence, histopathological findings, genetic background, diagnosis, and treatment. The present scoping review will allow us to identify the available evidence in the field, to bridge the gaps in the literature and to promote the improvement of interventions for the detection and management of patients with tubal EM.

## 2. Materials and Methods

### 2.1. Study Design and Eligibility Criteria

The present scoping review was designed in accordance with the guidelines for the Preferred Reporting Items for Systematic Reviews and Meta-Analyses extension for Scoping Reviews (PRISMA-ScR) according to the steps described by Tricco et al. [12]. All prospective and retrospective studies (comparative and non-comparative), case reports and case series that were written in English language and presented outcomes of patients with tubal EM were assessed and critically appraised. Experimental animal studies, letters to the editor, editorials, conference papers and reviews were excluded.

Our inclusion criteria were as follows: adult female patients age > 18 years; one or two fallopian tubes present to evaluate the characteristics of the presence of EM in the organ; tubal EM, which was histologically or macroscopically proved so as to ensure the examination of this certain pathology. Studies with reports on multiple EM sites were considered eligible provided that the tube was among the affected sites. History of salpingectomy and analysis of other pathologies of the fallopian tube except of the presence of EM were considered a criterion of exclusion from the study. The outcomes of interest were prevalence of tubal EM, disease-related characteristics and the genetic background of patients with tubal EM. 

### 2.2. Information Sources

A thorough and systematic search of the currently available literature was performed in three stages. Initially, three electronic databases (PubMed, Scopus and Google Scholar) were searched from January 2000 until August 2021. We identified a significant variation in the definition, distribution and contribution of tubal EM over the course of the years, and we have thus considered to include studies that have been published within the previous 20 years. The day of the last search was the 31st of August 2021. Titles and or abstracts of articles that presented outcomes on prevalence, characteristics and genetic profile of patients with tubal EM were evaluated for eligibility. Studies that were deemed to meet criteria were retrieved in full text. Finally, the references of the eligible articles were also searched for further identification of eligible studies. 

A minimum number of search keywords were utilized in an attempt to assess an eligible number of studies that could be easily searched while simultaneously minimizing the potential loss of articles. The following key words were utilized: “endometriosis”, “prevalence”, “tubal endometriosis”, “fallopian tube” and “endosalpingiosis”. 

### 2.3. Search

The search was performed using the keywords and Boolean operators. Our search strategy in PubMed used the following search terms:

- (“endometriosis” [MeSH Terms] OR “endometriosis” [All Fields] OR “endometrioses” [All Fields]) AND (“fallopian tubes” [MeSH Terms] OR (“fallopian” [All Fields] AND “tubes” [All Fields]) OR “fallopian tubes” [All Fields] OR (“fallopian” [All Fields] AND “tube” [All Fields]) OR “fallopian tube” [All Fields]);
- “tubal” [All Fields] AND (“endometriosis” [MeSH Terms] OR “endometriosis” [All Fields] OR “endometrioses” [All Fields]).

The PICO criteria that were used to develop our search strategy were as follows: 

Patient/Problem: Female adult patients suffering from tubal endometriosis, Intervention: Surgical evaluation of tubal endometriosis, Comparison: No tubal endometriosis, Outcome: Prevalence, disease-related characteristics, genetic background, diagnosis and treatment of tubal endometriosis. 

### 2.4. Selection of Sources of Evidence

The initial selection of articles was based on their title and then on the abstract in case of ambiguity for the eligibility of the study. After duplicates’ exclusion, the inclusion and exclusion criteria that are mentioned above were applied. Articles that fulfilled or were deemed to fulfil the inclusion criteria were retrieved. 

Three authors (AP, NK and DZ) independently and meticulously searched the literature, excluded overlaps, and tabulated the selected indices in structured forms. The discrepancy among the authors was discussed by all of them until they reached a consensus. 

### 2.5. Data-Charting Process, Data Items and Synthesis of Results

All authors discussed on the variables to be extracted by the included studies and structured tables that were independently fulfilled by two of them (AP and NK). After extraction, all authors discussed the validity of the extracted data and resolved potential discrepancies to achieve accuracy and validity. Data that was extracted included main study characteristics (country, study type, study period, patient number), patients’ characteristics, interventions and main outcomes. 

## 3. Results

### 3.1. Excluded Studies

A total of 3 studies were excluded from tabulation and analysis after reading their full text. More specifically, the study by Chakrabarti et al. was excluded as reported a case of EM that was developed in the fallopian stump four years after salpingectomy [13]. The studies by Sinha et al. and Audebert et al. did not present separate outcomes of patients with tubal EM apart from the prevalence of the disease among their study populations and were thus excluded [14,15].

### 3.2. Included Studies

A total of 13 studies were finally considered eligible for inclusion [16,17,18,19,20,21,22,23,24,25,26,27,28]. Among them, four were observational, which included a total of 633 patients and mainly focused on the prevalence of tubal EM among patients with various gynecological diseases, as well as on disease-related characteristics and histopathology [16,17,18,19], while two studies focused on analyzing the genetic profile of patients with tubal EM [20,21]. The remaining seven studies were case reports [22,23,24,25,26,27,28]. The main patient and disease characteristics of the included observational studies are shown in Table 1. A summary of the findings of the case reports is also depicted in Table 2. 

**Figure 1:**
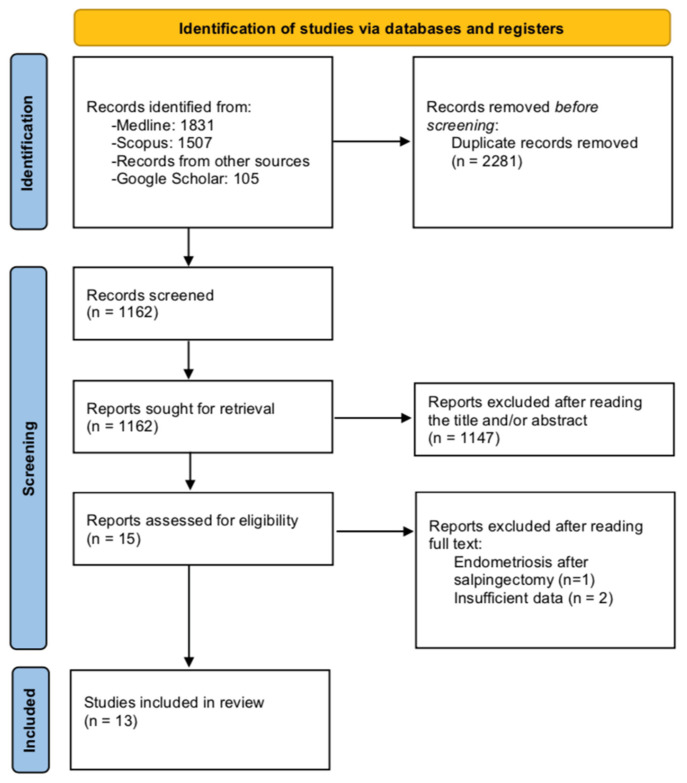
Search flow diagram.

The PRISMA search flow diagram schematically presents the stages of study selection and inclusion of the studies (Figure 1).

### 3.3. Prevalence and Disease Characteristics

Table 1 depicts the main study and patient characteristics derived from the included observational studies.

The prevalence of tubal EM ranged from 9% to 68.6% among the included studies. According to the prospective study by Xia et al., the prevalence of tubal EM in a group of 35 patients with pelvic EM was 68.6% (*n* = 24) [16]. In the study by Qi et al., 1112 premenopausal women who underwent salpingectomy due to various gynecological indications were grouped to those with and without tubal EM and analyzed [17]. In their study, the prevalence of tubal EM was 14.48% (*n* = 161/1112) [17]. The retrospective study by Xue et al. separated patients with tubal EM into three groups: those with EM (*n* = 178), those with other benign diseases (*n* = 65) and 18 others with malignant gynecologic diseases [18]. The prevalence of tubal EM was highest in the EM group. McGuinness et al. assessed the incidence of tubal EM among women who underwent operative laparoscopy due to EM, pelvic pain, infertility or adnexal cystic masses [19]. Ninety-seven patients underwent salpingectomy, whereas in 88 others, the macroscopic recognition of fallopian tube endometriotic lesions were ablated with CO_2_ laser, or electrosurgery (non-salpingectomy group). Tubal EM was detected in 35% (*n* = 34/97) and in 9% (*n* = 8/88) in the salpingectomy and non-salpingectomy groups, respectively, while the respective proportions in the subgroup of 153 patients with EM, was 42.5% for histologically proved tubal EM, and 11–12% for macroscopic tubal disease [19]. 

According to McGuinness et al., tubal EM was significantly related to severe disease when compared to mild or moderate (*p* = 0.0196) [19]. The same was also observed in the study by Qi et al., who reported an increment in tubal EM prevalence as the severity of pelvic EM increased (r = 0.26, *p* < 10^−4^) [17]. Regarding the factors that were related to elevated tubal EM rates, tubal ligation, abnormal uterine bleeding and previous surgery for EM were found significant in both uni- and multivariate analysis [17]. Additionally, patients with multi-organ EM presented an increased incidence of tubal EM compared to those with single-organ (43.94% vs. 24.24%, *p* < 0.05) [17]. 

Left side tubal EM was more prevalent than right side as proved by Qi et al. and Xue et al. (52.17% vs. 40.37%, *p* < 0.05 and *n* = 168/261, 64.37% vs. *n* = 93/ 261, 35.63%, *p* < 0.001, respectively) [17,18]. This was also observed when patients who were operated due to EM and malignant diseases were separately analyzed (*p* < 0.001 and *p* < 0.05, respectively) [18].

The literature search revealed a total of 7 case reports during the study period [22,23,24,25,26,27,28]. Table 2 depicts the main patients’ and disease-related characteristics from case reports. Median patients’ age was 33 years (range: 18–60), while five out of six patients were nulliparous. All patients were premenopausal except a case of detection of tubal EM in a 60-year-old postmenopausal woman who was diagnosed with clear-cell stage IIIC fallopian tube carcinoma associated with an endometriotic tubal wall cyst. Only one patient reported a history of EM prior to surgery. 

### 3.4. Histopathological Findings

The analysis of patients by Xia et al. revealed significantly decreased ciliary beat frequency (CBF) in both ampulla and isthmus when compared to either 20 control patients who underwent surgery for uterine leiomyoma or the remaining 11 without EM (non-tubal EM group) [16]. The same was also observed in the percentages of ciliated cells. Finally, tubal EM group presented significantly lower contraction frequencies and weaker muscular contractility [16]. Concerning the histopathological findings reported by Qi et al., mucosa and serosa were the most common layers of tubal EM detection with more than 80% of the proximal tubal lesions detected in the mucosa, whereas 53.85% of lesions in the distal tube were found in the serosa [17]. Finally, serosal lesions presented a more prominent inflammatory reaction and fibroblasts and collagenous proliferation near the lesion than mucosal ones [17]. 

### 3.5. Genetic Background

The study group by Qi et al. recently published two studies on the analysis of the genetic profile of tubal EM [20,21]. More specifically, a study published in 2019 compared the miRNA-microarray expression among four patients with tubal EM and five controls [20]. The authors identified a total of 17 miRNAs in the tubal epithelium that were expressed different in the tubal EM group (four upregulated and 13 downregulated) [20]. Bioinformatic analysis revealed that some of the detected miRNAs play a significant role in the mTOR signaling pathway, SNARE interactions and endocytosis, thus participating in the pathogenesis of EM [20]. Accordingly, a study published in 2020 by the same study group found a total of 50 significantly dysregulated genes in the tubal epithelial analysis of four women with tubal EM compared to specimens of four controls without tubal EM, while a respective proteomic analysis of tubal fluid showed 33 over-expressed proteins and 19 under-expressed ones in patients with tubal EM [21]. Among them, IL-6, TNFA, C2, C4B, MMP7 and AHSG are common proteins that were found to be preferentially expressed in patients with tubal EM both in epithelium and tubal fluid [21]. Additionally, ORM2, SAA4, CP HP and MAP2K6 are some further innovative proteins that have also been identified [21]. IL-6, C4B, CP, C2, HP, TNFA and ORM2 were among the up-regulated proteins while AHSG and MAP2K6 were the down-regulated ones [21]. The commonly expressed genes and proteins participated in the inflammatory response, cellular movement and immune cell trafficking, which can all explain a part of the molecular mechanisms of EM formation [21]. 

### 3.6. Diagnosis

According to the data derived from case reports, the primary indication for surgery was infertility in three patients. Among them, two had primary infertility and one was a primiparous patient with secondary infertility. The case reported by Kahyaoglou et al., suffered from 18-year infertility and presented with acute pelvic pain and vaginal bleeding 20 days after embryo transfer, and thus referred to emergent laparoscopy with the suspicion of ectopic pregnancy [24]. Acute abdominal pain was also the predominant symptom in two patients who underwent emergent surgery, whereas two other patients reported dull abdominal pain and distention. The preoperative imaging findings and the reported histopathological findings are shown in Table 2. 

### 3.7. Treatment-Follow-Up

All seven patients from the cases reports underwent surgery for the management of their disease. Intraoperative findings revealed that among the five patients with no previous EM history, EM implants were identified during surgery in four of them, while in one patient no intraoperative EM lesions were macroscopically detected. The last patient underwent surgery for suspected hydrosalpinx, and no EM signs were present at macroscopic examination during diagnostic laparoscopy, while histological examination of the excised right tube revealed intraluminal tubal EM. Five patients had laparoscopic approach and the remaining two underwent laparotomy. In four patients the tubal EM lesion was right-sided, in one a left tubal EM was detected and two other had bilateral EM tubal lesions. Five patients aged from 18 to 34 years underwent salpingectomy to manage their disease, whereas two patients aged 49 and 60 years underwent total abdominal hysterectomy (TAH) with right salpingo-ophorectomy, and (TAH) with bilateral salpingo-ophorectomy, pelvic lympadenectomy and para-aortic lympadenectomy, respectively. From the five patients that had salpingectomy, follow-up was available for two of them with both being disease-free with no evidence of EM recurrence at follow up [22,23]. 

## 4. Discussion

Despite the extensive research on the characteristics and prevalence of both ovarian EM and other EM sites either pelvic or extrapelvic, data on tubal EM as an independent entity still remain limited. We sought to investigate the contribution of the fallopian tubes to the formation and pathogenesis of EM and the unique characteristics of the disease. The outcomes of the present study revealed a variable prevalence of tubal EM that ranged from 6.9% to 69%. However, this should be interpreted with caution based on the heterogeneous populations of the included studies that enrolled patients with concomitant pelvic EM, others with incidental detection of EM during surgery for other indications (benign or malignant) or women suffering from pain or infertility. The true prevalence of tubal EM remains elusive. Data from case reports showed that the main symptom for referral of patients with tubal EM was infertility and abdominal pain, while as high as 71% of patients did not report a previous EM history. The management of the disease in women of reproductive age included salpingectomy.

Preoperative diagnosis of tubal EM is challenging due to the lack of disease-specific ultrasonographic characteristics. It can be recognized as hematosalpinx, hydrosalpinx or sactosalpinx with the transvaginal ultrasound [29]. Stepniewska et al. reported that ultrasonographic detection of hydrosalpinx could be suggestive of the presence of tubal EM with specificity as high as 99%, but low specificity of 12%, which could potentially be improved by incorporating other diagnostic markers [29]. 

Based on the findings from the included studies, tubal EM can be recognized in all tubal layers and parts, with the most common being the mucosal proximal and serosal distal lesions. Notably, three different histological subtypes of tubal EM have been described; the first one refers to lesions identified in the serosa of subserosa of the tube and can be macroscopically recognized as implants even in the peritoneal tubal surface [30]. This type can mainly cause hydrosalpinx due to the endometriosis associated fibrosis and the retraction of the tube. The second subtype is recognized in the tubal mucosa. It can be caused by the development of ectopic endometrium in the tubal lumen and results in obstruction and hematosalpinx of the endometriotic implants due to the cyclical hemorrhage. However, about 9% in the study by Qi et al. had lesions located in both mucosa and serosa [17]. Finally, the development of EM in the tubal stump post-salpingectomy has been reported and is the third distinct type reported by a limited number of studies [13]. 

The presence of tubal EM could be implicated in the pathogenesis of women with infertility. The tubal-related infertility is associated with tubal dysfunction that is most commonly caused by obstruction, adhesions and hydrosalpinx. Tubal obstruction and adhesions account for approximately 20% of cases of subfertility [30]. In the case of tubal EM, the pathology mentioned above is also complicated by the inflammatory response. This is mediated by the presence of endometrial implants and promotes the secretion of cytokines/growth factors/chemokines and concentration of macrophages which could be the reason for the endometriotis-related pain [31]. This is also proved by the identification of proteins such as IL-6 that are involved in the immune response in genetic analysis of the specimens excised from patients with tubal EM [21]. The involvement of endometrial stem/progenitor stem cells in the pathogenesis and development of EM has also gained significant popularity [32]. Those cells are normally identified in regenerated endometrium [32]. However, they can enter the pelvis through retrograde menstruation and under unknown conditions trigger the formation of endometrial glands and stroma in sites outside the uterus which is compatible with EM [32]. Furthermore, apart from tubal obstruction, tubal EM has been claimed to interfere to the ciliary function and normal muscle contraction of the tube as also reported in our results creating an unfriendly environment for the transportation of the sperm and the subsequent insemination [16,30]. As a result, the effective management of tubal EM is of critical clinical importance. The trends in the management of tubal EM during the course of the years could not be precisely estimated due to the limited available data while in a significant proportion of patients the identification of the disease is incidental during salpingectomy for the management of other pathologies. According to the data derived from case reports of patients with suspected tubal EM, surgical management still remains the gold standard of therapy.

Our outcomes indicate a predominance of left-sided tubal EM lesions and are compatible with some reports on the asymmetrical distribution of the disease in the pelvic sides [17,18]. Similarly, Sznurkowski et al. found significantly increased left-sided endometriomas compared to right-sided in patients with unilateral disease (odds ratio 2.8, 95% CI 1.9 to 4.4, *p* < 0.001) [33]. Various theories have sought to explain the asymmetry of the disease mainly based on the anatomical differences of each hemipelvis. In particular, the exfoliation of endometrial cells that are in the peritoneal cavity during menstruation through the tubes is less feasible on the left side due to the presence of the sigmoid colon [34]. Additionally, the peritoneal fluid has been reported to flow slower in the left hemipelvis, and as a result the left hemipelvis could be considered more susceptible to the effect of menstrual endometrial fragments. [34]. The left lower part of the pelvis also represents the final site in the clockwise flow of the peritoneal fluid [35]. Lastly, another theory supports that the left hemipelvis is under the lower effect of progesterone, which is a significant regulator of the endometrial tissue, due to the more elevated levels of the hormone produced by the right ovary, which ovulates more frequent than the left one [34]. Moreover, the transportation theory is supportive of the fact that the left sided predominance in the tube could be a bridge for the transportation of endometriotic tissue to the left ovary and left endometrioma [18]. It is obvious that no safe conclusion regarding the most dominant theory of the left-side predominance of tubal EM can be drawn due to the heterogeneity of the available studies.

Our search revealed a case of clear-cell malignant histology associated with a tubal endometriotic cyst [28]. Unfortunately, no data was available on the long-term survival outcomes of this patient. The theory of initiation of some histological types of ovarian cancer from fallopian tubes has recently gained significant popularity. In particular, except for the serous histological subtype, there is strong evidence of the fallopian origin of some of endometrioid and clear cell ovarian carcinomas [11, 36]. Moreover, the risk of malignant transformation in patients with endometriosis has been reported, mostly related to endometrioid and clear cell histology [8]. In fact, it seems that ovarian endometriosis may be associated to ovarian malignancies by sharing some pathophysiological pathways [11]. In particular, a variety of genetic mutations including PTEN, BRACA1, BRACA2 and KRAS have been detected in both pathologies. Furthermore, they have also found to share some miRNA alterations that interfere with genetic expression [11]. The correlation between endometriosis and malignant transformation and the potential fallopian origin of those pathologies should not be neglected. Consequently, further research is needed in the field so as to elucidate the exact interaction among cell differentiation and development of cancer as well as the cellular source, which could all have a significant clinical impact given the ability to perform risk reducing procedures in patients that could be considered as high risk for developing malignancy. 

### Limitations of the Study

A number of inherent limitations need to be addressed. The limited number of existing studies and as a consequence the small number of the included patients and the significant heterogeneity in eligibility criteria cannot allow us to reach any instrumental conclusion. Despite the broad variation from 6.9% to 69% among the included studies on the prevalence of tubal EM, this cannot allow us to draw any safe conclusion on the exact prevalence of the disease due to the heterogeneity of the included study populations from each study. Nonetheless, our study aims to critically review an underexplored condition that impacts on a broad spectrum of gynecologic problems from infertility to ovarian cancer. The majority of the results were derived from observational studies by the same study group from Asia. However, different parameters are evaluated by each one of them as shown in Table 1. The fact that the we chose to include studies that have been published within the previous 20 years was based on the differences in the definition, distribution and contribution of tubal EM that have been reported in the course of the years. Despite the fact that the first report on patients with tubal EM dates back in 50s who described three cases of women with tubal endometriotic lesions in the isthmian tubal part, tubal involvement in EM was underestimated [37]. The majority of the studies focused on the prevalence and characteristics of the disease and lack of fertility and postoperative outcomes regarding pain control. Therefore, we considered analyzing the data from case reports so as to assess the preoperative characteristics and management of patients with suspected tubal EM. 

## 5. Conclusions

Tubal EM is a distinct entity with prevalence that ranges depending on the indication for surgery, presence of concomitant pelvic EM and the type of diagnosis and treatment. The analysis of the genetic profile of patients with tubal EM could decipher the molecular pathways of EM formation and evaluate the exact role of the inflammatory, cellular and immune response so as to designate and individualize the treatment. The identification of molecular targets and pathways in tubal EM could also enable the application of personalized therapy. Further larger population-based studies are needed in the field so as to evaluate the impact of tubal EM in the progression and prognosis of EM, the effect of salpingectomy in the improvement of the disease-related symptoms and to define the group of patients that could benefit from risk-reducing salpingectomy based on the risk of developing ovarian malignancy.

## Figures and Tables

**Table 1:** Characteristics of the included observational studies and patients.

*Year; Author*	*2018; Xia*	*2019; Qi*	*2020; Xue*	*2020; Mcguinness*
***Country***	China	China	China	USA
***Type of study***	PS	Cross-sectional	RS	RS
***Study period***	06/2016–08/2017	06/2016–08/2017	01/2002–07/2019	07/2015–06/2018
***Inclusion criteria***	Patients with uterine leiomyoma and adenomyosis treated with hysterectomy and salpingectomy; no hormonal medication within 3 mo; no history of tubal surgery	Premenopausal; unilateral or bilateral salpingectomy; complete data; no pregnancy; consent for participation	Salpingectomy	Surgery for EM by MIS; age < 55; no malignant cases; no previous laparotomy; no previous bil salpingectomy
***Main outcomes***	Ciliary beat frequency (CBF)	Characteristics, prevalence, clinical features, pathologic features, predictors of EM	Prevalence of tubal EM among groups	Prevalence of tubal EM among groups
***Compared groups***	AM without EM vs. EM without AM vs. control (uterine leiomyoma)	EM vs. no EM	EM vs. BN vs. MT	Salpingectomy vs. no salpingectomy
***Indication for surgery***	Leiomyoma, AM, EM	Fibroid, ovarian cyst, salpingitis/infertility, hydrosalpinx, malignancy, tubal sterilization, adenomyosis, EM	Leiomyoma, adenomyosis, endometrioid cysts, hydrosalpinx, uterine malformation, malignancy	EM, pelvic pain, cystic adnexal mass, infertility, fibroids, AUB
***Patients (n)***	75 (20 vs. 35 vs. 20)	1112 (161 vs. 951)	261 (178 vs. 65 vs. 18)	185 (97 vs. 88)
***Patients age (years)***	44.4 ± 5.2 ^a^ vs. 43.4 ± 5.1 ^a^ vs. 47.2 ± 4.8 ^a^ (AM vs. EM vs. control)	44.89 ± 6 ^a^ vs. 45.9 ± 5.97 ^a^, *p* = 0.002 (tubal EM vs. no EM)	44 ± 7 ^a^ (total)	41.26 ± 7.45 ^a^ vs. 34.24 ± 7.37 ^a^ (salpingectomy vs. no salpingectomy)
***Other EM sites***	N/A	Ovarian EM L: 70/R: 53/Bil: 34	Ovarian EM L: 70/R: 49	N/A
***Site of EM (L/R/Bil)***	N/A	84 (40.37%)/65 (52.17%)/12 (7.45%), *p* < 0.005 (for L/R)	168 (55.08%)/93 (30.49%)/44 (14.43%), *p* < 0.001 (for L/R)	N/A
***Prevalence of tubal EM***	24/35 (69%) for EM group	161/1112 (14.48%)	EM group: 178 (68.2%) BN group: 65 (24.9%) MT group: 18 (6.9%)	34/97 (35%) salpingectomy group vs. 8/88 (9%) no salpingectomy group
***Location in tube (tubal site/histologic layer)***	N/A	Proximal: 78 (48.45%) Distal: 78 (48.45%) Proximal + distal: 5 (3.1%)/ Mucosa: 88 (54.66%) Myosalpinx: 10 (6.21%) Serosa: 52 (32.3%) Mucosa + serosa: 11 (6.83%)	N/A	N/A
***Predisposing factors***	N/A	Previous EM, multi-organ EM, uterine seromuscular EM, severity of pelvic EM, young age, AUB, previous tubal ligation	N/A	N/A

RS: retrospective, EM: endometriosis, AM: adenomyosis, BN: benign disease, MT: malignant disease, MIS: minimally invasive surgery, AUB: abnormal uterine bleeding, PID: pelvic inflammatory disease, IUD: intrauterine device, L: left, R: right, Bil: bilateral, ^a^ Mean ± SD, N/A: not available.

**Table 2:** Main characteristics of patients from case reports.

*Year; Author*	*Age (Years)*	*Primary Symptom*	*Parity*	*Imaging Findings*	*Pre-Surgical Diagnosis (Indication for Surgery)/Operative Procedure-Findings*	*Menopausal Status*	*History of EM/IO EM Findings*	*Histological Findings*	*Side/Site of Tubal EM*
2013; Wenger	18	Acute pelvic pain, oligomenorrhea, persistent dysmenorrhea and dyspareunia	Nulli	TVUS: hypoechoic structure 13 × 10 in the rectovaginal septum, MRI: oval-shaped nodule 30 × 20 mm hypertense structure on T1, hemoglobin products in T2	DIE/DL-multiple red, black, and white scarred EM implants in uterosacral ligaments, R tubal cyst, fallopian tube torsion, R distal portion salpingectomy and adhesiolysis	Pre	No/EM implants identified during surgery	Tubal endometrioma with multiple sclerotic and calcified areas, stroma cells and hemosiderin-laden macrophages	R distal portion
2012; Lim	30	5 month dysmenorrhea and dull lower abdominal pain	Nulli (virgin)	Thick-walled, complex cystic structures 21 × 21 mm and 53 × 34 mm (R and L ovary)	Pelvic EM/DL-bilateral torted tubes and cystic dilation at the distal portion salpingectomy and adhesiolysis	Pre	No/EM implant (spot) identified during surgery	Extensive hemorrhagic infarction secondary to torsion and hematosalpinx with endometrial glands detection	Bilateral distal portion
2011; Kahyaoglu	33	18 years infertility and mild EM, pelvic pain and vaginal bleeding after embryo transfer	Nulli	TVUS: R tubal ectopic ring	Ectopic pregnancy/Emergent laparoscopy- bilateral salpingectomy	Pre	Yes (pelvic peritoneum)	Bilateral tubal ectopic pregnancy with endometriotic implants	Bilateral
2010; Ozturk	31	Secondary infertility	Primi	TVUS: R hydrosalpinx 37 × 12 mm	Hydrosalpix/DL-dilated R tubal uterine mimicking hydrosalpinx, R salpingectomy	Pre	No/No IO EM implants	Intraluminal tubal EM	R mucosa
2004; Datta	34	Primary infertility	Nulli	TVUS: Polycystic ovaries, HSG: normal	Unexplained infertility/DL -atypical endometriotic deposit on R tube mimicking ectopic pregnancy, ovarian drilling	Pre	No/EM uterosacral implants identified during surgery	Not performed	R
2003; Ohara	49	Anemia, acute abdominal pain	Nulli	US: R elongated sausage-shaped cystic mass 6.2 × 3.3 cmm, CA 125: 57.7 U/mL	Hematosalpinx/Emergent laparotomy-R elongated distended dark purple tube with occluded fimbrial end triple twisted, TAH-RSO	Pre	No/EM implants identified during surgery	Extensive hemorrhagic infarction secondary to torsion and endometrial glands in the haematosalpinx	R
2002; De la Torre	60	Abdominal distension and pelvic pain	N/A	US: Tumor with solid and cystic components 10 cm, CT: L para-aortic node 1 cm	Ovarian cancer/Exploratory laparotomy- TAH BSO PL PaL	Post	N/A	Transitional areas between the newly formed and endometriotic epithelium lined the cystic cavity of tubal wall-Clear cell fallopian tube carcinoma with tubal EM	L-proximal portion 1 cm from uterine ostium

N/A: not available, DL: diagnostic laparoscopy, TVUS: transvaginal ultrasound, DIE: deep infiltrating endometriosis, R: right, L: left, EM: endometriosis, HSG: hysterosalpingography, TAH: total abdominal hysterectomy, RSO: right salpingoophorectomy, BSO PL Pal: bilateral salpingoophorectomy pelvic lympadenectomy and para-aortic lympadenectomy.

## Data Availability

Upon request.

## References

[1] Gruber T.M., Mechsner S. (2021). Pathogenesis of Endometriosis: The Origin of Pain and Subfertility. Cells.

[2] Taylor H.S., Kotlyar A.M., Flores V.A. (2021). Endometriosis is a chronic systemic disease: Clinical challenges and novel innovations. Lancet.

[3] Lee H.J., Park Y.M., Jee B.C., Kim Y.B., Suh C.S. (2015). Various anatomic locations of surgically proven endometriosis: A single-center experience. Obstet. Gynecol. Sci..

[4] Chapron C., Fauconnier A., Vieira M., Barakat H., Dousset B., Pansini V., Vacher-Lavenu M.C., Dubuisson J.B. (2003). Anatomical distribution of deeply infiltrating endometriosis: Surgical implications and proposition for a classification. Hum. Reprod..

[5] Ávalos Marfil A., Barranco Castillo E., Martos García R., Mendoza Ladrón de Guevara N., Mazheika M. (2021). Epidemiology of Endometriosis in Spain and Its Autonomous Communities: A Large, Nationwide Study. Int. J. Environ. Res. Public Health.

[6] Gordts S., Koninckx P., Brosens I. (2017). Pathogenesis of deep endometriosis. Fertil. Steril..

[7] Arafah M., Rashid S., Akhtar M. (2021). Endometriosis: A Comprehensive Review. Adv. Anat. Pathol..

[8] Giannella L., Marconi C., Di Giuseppe J., Delli Carpini G., Fichera M., Grelloni C., Giuliani L., Montanari M., Insinga S., Ciavattini A. (2021). Malignant Transformation of Postmenopausal Endometriosis: A Systematic Review of the Literature. Cancers.

[9] Pearce C.L., Templeman C., Rossing M.A., Lee A., Near A.M., Webb P.M., Nagle C.M., Doherty J.A., Cushing-Haugen K.L., Wicklund K.G. (2012). Association between endometriosis and risk of histological subtypes of ovarian cancer: A pooled analysis of case-control studies. Lancet. Oncol..

[10] de la Cuesta R.S., Eichhorn J.H., Rice L.W., Fuller A.F., Nikrui N., Goff B.A. (1996). Histologic transformation of benign endometriosis to early epithelial ovarian cancer. Gynecol. Oncol..

[11] Gaia-Oltean A.I., Braicu C., Gulei D., Ciortea R., Mihu D., Roman H., Irimie A., Berindan-Neagoe I. (2021). Ovarian endometriosis, a precursor of ovarian cancer: Histological aspects, gene expression and microRNA alterations (Review). Exp. Ther. Med..

[12] Tricco A.C., Lillie E., Zarin W., O’Brien K.K., Colquhoun H., Levac D., Moher D., Peters M.D.J., Horsley T., Weeks L. (2018). PRISMA Extension for Scoping Reviews (PRISMA-ScR): Checklist and Explanation. Ann. Intern. Med..

[13] Chakrabarti I., Ghosh N. (2010). Post-salpingectomy endometriosis: An under-recognized entity. J. Mid-Life Health.

[14] Audebert A., Petousis S., Margioula-Siarkou C., Ravanos K., Prapas N., Prapas Y. (2018). Anatomic distribution of endometriosis: A reappraisal based on series of 1101 patients. Eur. J. Obstet. Gynecol. Reprod. Biol..

[15] Sinha A.K., Agarwal A., Lakhey M., Mishra A., Sah S.P. (2003). Incidence of pelvic and extrapelvic endometriosis in Eastern region of Nepal. Indian J. Pathol. Microbiol..

[16] Xia W., Zhang D., Ouyang J., Liang Y., Zhang H., Huang Z., Liang G., Zhu Q., Guan X., Zhang J. (2018). Effects of pelvic endometriosis and adenomyosis on ciliary beat frequency and muscular contractions in the human fallopian tube. Reprod. Biol. Endocrinol. RBE.

[17] Qi H., Zhang H., Zhang D., Li J., Huang Z., Zhao X., Zhang J. (2019). Reassessment of prevalence of tubal endometriosis, and its associated clinicopathologic features and risk factors in premenopausal women received salpingectomy. Eur. J. Obstet. Gynecol. Reprod. Biol. X.

[18] Xue R.H., Li J., Huang Z., Li Z.Z., Chen L., Lin Q., Huang H.F. (2020). Is tubal endometriosis an asymmetric disease? A 17-year retrospective study. Arch. Gynecol. Obstet..

[19] McGuinness B., Nezhat F., Ursillo L., Akerman M., Vintzileos W., White M. (2020). Fallopian tube endometriosis in women undergoing operative video laparoscopy and its clinical implications. Fertil. Steril..

[20] Qi H., Liang G., Yu J., Wang X., Liang Y., He X., Feng T., Zhang J. (2019). Genome-wide profiling of miRNA expression patterns in tubal endometriosis. Reproduction.

[21] Qi H., Zhang H., Zhao X., Qin Y., Liang G., He X., Zhang J. (2020). Integrated analysis of mRNA and protein expression profiling in tubal endometriosis. Reproduction.

[22] Wenger J.M., Soave I., Lo Monte G., Petignat P., Marci R. (2013). Tubal endometrioma within a twisted fallopian tube: A clinically complex diagnosis. J. Pediatric Adolesc. Gynecol..

[23] Lim S.Y., Park J.C., Bae J.G., Kim J.I., Rhee J.H. (2012). Isolated torsion of bilateral fallopian tubes combined with tubal endometriosis: A case report. Korean J. Obstet. Gynecol..

[24] Kahyaoğu İ., Kahyaoğlu S., Müftüoğlu K., Şengül İ., Şengül D., Önen Ş., Mollamahmutoğlu L., Batıoğlu S. (2011). Tubal endometriosis as an insidious risk factor for tubal implantation in a procedure of assisted reproductive technique. Cumhur. Med. J..

[25] Ozturk E., Ugur M., Aydın A., Balat O., Kalaycı H. (2010). Intraluminal tubal endometriosis mimicking hydrosalpinx: Report of an unusual case. IIUM Med. J. Malays..

[26] Datta S., Priddy A. (2004). Tubal endometriosis mimicking an ectopic pregnancy. Reproduction.

[27] Ohara N., Narita F., Murao S. (2003). Isolated torsion of haematosalpinx associated with tubal endometriosis. J. Obstet. Gynaecol. J. Inst. Obstet. Gynaecol..

[28] de la Torre F.J., Rojo F., García A. (2002). Clear cells carcinoma of fallopian tubes associated with tubal endometriosis. Case report and review. Arch. Gynecol. Obstet..

[29] Stepniewska A.K., Clarizia R., De Mitri P., Pesci A., Zorzi C., Albanese M., Trivella G., Guerriero M., Improda F.P., Ceccaroni M. (2021). Role of ultrasonographic parameters for predicting tubal involvement in infertile patients affected by endometriosis: A retrospective cohort study. J. Gynecol. Obstet. Hum. Reprod..

[30] Hill C.J., Fakhreldin M., Maclean A., Dobson L., Nancarrow L., Bradfield A., Choi F., Daley D., Tempest N., Hapangama D.K. (2020). Endometriosis and the fallopian tubes: Theories of origin and clinical implications. J. Clin. Med..

[31] Garcia-Velasco J.A. (2020). Fallopian tube endometriosis: Clinical implications. Fertil. Steril..

[32] Sznurkowski J.J., Emerich J. (2008). Endometriomas are more frequent on the left side. Acta Obstet. Gynecol. Scand..

[33] Araujo R., Maia S.B., Lúcio J.D., Lima M.D., Ribeiro H., Ribeiro P. (2021). Mapping of endometriosis in patients with unilateral endometrioma. Medicine.

[34] Scioscia M., Bruni F., Ceccaroni M., Steinkasserer M., Stepniewska A., Minelli L. (2011). Distribution of endometriotic lesions in endometriosis stage IV supports the menstrual reflux theory and requires specific preoperative assessment and therapy. Acta Obstet. Gynecol. Scand..

[35] Wang Y., Mang M., Wang Y., Wang L., Klein R., Kong B., Zheng W. (2015). Tubal origin of ovarian endometriosis and clear cell and endometrioid carcinoma. Am. J. Cancer Res..

[36] Revzin M.V., Moshiri M., Katz D.S., Pellerito J.S., Mankowski Gettle L., Menias C.O. (2020). Imaging Evaluation of Fallopian Tubes and Related Disease: A Primer for Radiologists. RadioGraphics.

[37] Madsen V. (1943). The salpingographic picture in tubal endometriosis. Acta Obstet. Gynecol. Scand..

